# iTex Gloves: Design and In-Home Evaluation of an E-Textile Glove System for Tele-Assessment of Parkinson’s Disease

**DOI:** 10.3390/s23062877

**Published:** 2023-03-07

**Authors:** Vignesh Ravichandran, Shehjar Sadhu, Daniel Convey, Sebastien Guerrier, Shubham Chomal, Anne-Marie Dupre, Umer Akbar, Dhaval Solanki, Kunal Mankodiya

**Affiliations:** 1 Department of Electrical, Computer and Biomedical Engineering, University of Rhode Island, Kingston, RI 02881, USA; 2 Department of Physical Therapy, University of Rhode Island, Kingston, RI 02881, USA; 3 Department of Neurology, Brown University, Rhode Island Hospital, Providence, RI 02903, USA

**Keywords:** Internet of Things, telemedicine, e-textiles, wearable sensors, smart gloves, Parkinson’s disease

## Abstract

Parkinson’s disease (PD) is a neurological progressive movement disorder, affecting more than 10 million people globally. PD demands a longitudinal assessment of symptoms to monitor the disease progression and manage the treatments. Existing assessment methods require patients with PD (PwPD) to visit a clinic every 3–6 months to perform movement assessments conducted by trained clinicians. However, periodic visits pose barriers as PwPDs have limited mobility, and healthcare cost increases. Hence, there is a strong demand for using telemedicine technologies for assessing PwPDs in remote settings. In this work, we present an in-home telemedicine kit, named iTex (**i**ntelligent **Tex**tile), which is a patient-centered design to carry out accessible tele-assessments of movement symptoms in people with PD. iTex is composed of a pair of smart textile gloves connected to a customized embedded tablet. iTex gloves are integrated with flex sensors on the fingers and inertial measurement unit (IMU) and have an onboard microcontroller unit with IoT (Internet of Things) capabilities including data storage and wireless communication. The gloves acquire the sensor data wirelessly to monitor various hand movements such as finger tapping, hand opening and closing, and other movement tasks. The gloves are connected to a customized tablet computer acting as an IoT device, configured to host a wireless access point, and host an MQTT broker and a time-series database server. The tablet also employs a patient-centered interface to guide PwPDs through the movement exam protocol. The system was deployed in four PwPDs who used iTex at home independently for a week. They performed the test independently before and after medication intake. Later, we performed data analysis of the in-home study and created a feature set. The study findings reported that the iTex gloves were capable to collect movement-related data and distinguish between pre-medication and post-medication cases in a majority of the participants. The IoT infrastructure demonstrated robust performance in home settings and offered minimum barriers for the assessment exams and the data communication with a remote server. In the post-study survey, all four participants expressed that the system was easy to use and poses a minimum barrier to performing the test independently. The present findings indicate that the iTex glove system has the potential for periodic and objective assessment of PD motor symptoms in remote settings.

## 1. Introduction

Parkinson’s disease (PD) is a progressive neurodegenerative disorder that impacts nearly 10 million individuals worldwide [1]. PD is characterized by various movement symptoms including akinesia (inability to perform movement), bradykinesia (slowness of movement), rigidity (muscles contracting involuntarily), tremors, and postural instability. Present interventions can reduce certain movement symptoms, but they do not halt or reverse the disease progression [2]. Therefore, individuals require longitudinal evaluation of movement and non-movement symptoms to assess the changes in movement symptoms associated with PD progression and/or medications [3]. These periodic movement symptom assessments are used by neurologists to adjust therapeutic intervention.

The Movement Disorder Society Unified Parkinson’s Disease Rating Scale (MDS-UPDRS) was proposed to enable clinicians to assess symptoms associated with PD. The scale consists of four parts that evaluate non-movement and movement symptoms in daily living conditions while performing certain upper-body and lower-body movement tasks [4]. The third part of the MDS-UPDRS examination is designated to evaluate the progression of movement symptoms through visual inspection by a neurologist while PwPD perform certain repetitive movement tasks. Those tasks include but are not limited to forearm pronation/supination, finger taps, hand open–close, and finger-to-nose movement.

The UPDRS Part III evaluation is time-consuming and inefficient for both the PwPD and neurologists resulting in large intervals between assessment periods. Studies have also shown that UPDRS raters can have their assessment influenced by their individual clinical experiences, resulting in variability between raters [5]. The highly episodic nature of the PD symptoms and the brief doctor’s visit can cause the evaluator to misjudge or misclassify symptoms. Therefore, it is imperative to have technological solutions, enabling periodic movement assessments in daily life settings.

**Figure 1:**
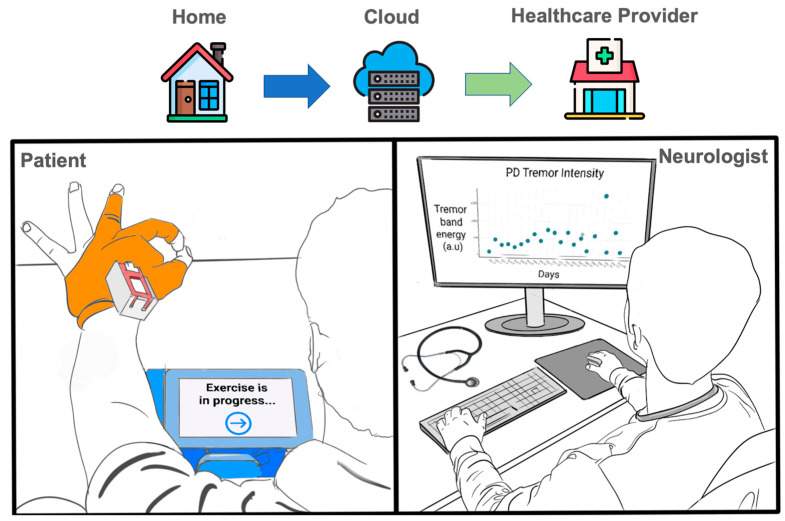
An overview of the iTex system (**left**). User performing movement exams while wearing iTex gloves at home. Clinician reviewing movement symptom analytics over a period from the clinic (**right**).

To meet this need, our presented research is aimed at designing and evaluating a smart textile system, named iTex (**i**ntelligent **Tex**tile), for in-home movement assessments using connected smart gloves (i.e., iTex gloves) and patient interface tablet as seen in Figure 1. iTex offers an unobtrusive modality using clothing to collect bodily data in a longitudinal context through the integration of sensors and wireless devices within textile materials.

The presented research work makes core contributions as follows:

1. Smart Textiles—iTex Gloves: Sensing gloves are specially engineered to acquire fine and gross motor symptoms through finger flexion and inertial signals for movement in daily living conditions. iTex gloves require no expensive infrastructure such as cameras. The inclusion of flexion sensing enables the assessment of upper body movement tasks that are found in the standard protocols such as UPDRS Part III. The gloves are designed with the consideration of ease of use for accommodating the movement impairments of PD.
2. Wearable IoT System: The IoT system connects with the gloves and displays the patient interface. It ensures the orchestration of the motor assessment delivery that requires on-demand data acquisition of movement data using WiFi-based MQTT protocol.
3. Patient-Centered Exercise Application: A patient-centered user interface was developed to guide PwPD through the movement screening tasks, perform data collection of the gloves, and deliver the daily questionnaires. Section 3 offers more details on the various components of the application from the user interface, flask server, IoT data acquisition, time-series database, and cloud export.
4. In-Home Evaluation of the iTex Glove with PwPD: A feasibility study was conducted in real-world home environments. Four PwPDs were recruited to use the iTex system independently two times a day for a week. Real-world data throughput, adherence, and timing of different system operations are studied for the proposed system. To examine the usability constraints of the system with PwPD, a brief exit interview was conducted.
5. Signal Processing, Feature Analysis, and Medication Status Classification: Features specific to kinetic movement tasks and stationary tasks were extracted from inertial (pitch, roll) and index flexion signals. Preliminary analysis of movement assessment activity data indicates variability in signal features based on medication usage states. Performance of different machine learning classification models for the feature dataset is reported along with SHAP-based feature importance scores.

## 2. Background

- Parkinson’s disease and its management

The pathology of PD includes movement and non-movement symptoms that functionally limit people with PwPD. This work primarily looks to evaluate movement symptoms associated with PD primarily in the upper body. The movement symptoms associated with PD in the upper body include but are not limited to Parkinsonian tremors, dyskinesia, and bradykinesia [2]. Parkinsonian tremors are characterized by a slow rhythmic high-amplitude movement during rest with a frequency range of 3–10 Hz [6]. With the eventual progression of PD symptoms, a medication known as Levodopa is prescribed to patients [7]. The medication gets metabolized into dopamine within the central nervous system which reduces or alleviates the movement symptoms associated with PD. The medication’s effect lasts for a certain period (half-life 2.5 hrs [7]), and its effectiveness is based on a multitude of factors such as medication type (extended-release vs. regular), physiological factors (sleep and stress [8]), and PD progression and severity. However, this medication has a side effect that can manifest as uncontrolled involuntary movements known as dyskinesias [9]. The dyskinesia symptoms are often mild and bearable leading PwPD to predominantly prefer those symptoms over parkinsonian movement symptoms [10]. However, dyskinesia symptoms might significantly interfere with daily living in certain PwPD requiring adjustment of the medication plan.

B. Telemedicine and its role in PD symptom management

Telemedicine refers to the utilization of technology to provide remote medical care, such as follow-up medical appointments or physical therapy [11]. Telemedicine in the context of PD holds particular significance due to the progressive nature of the ailment combined with long intervals between evaluation sessions. Here, we will discuss various solutions to address movement assessments of PD. Symptom diaries enable PwPD to track self-reported movement and non-movement symptoms by filling out daily questionnaires tracking the severity of various symptoms [12]. However, these diaries are subjective in nature and do not correlate with expert assessment or data from wearables, resulting in false negatives and false positives for self-reported measures of the presence/absence of dyskinesia or tremors [13]. Optical movement assessment is a method that encompasses a wide range of technology, from marker-based tracking (used in performance motion capture) to marker-less vision-based tracking (infrared and visible imaging). Marker-based tracking methods seek to evaluate pose, movement, and gait by utilizing infrared or reflective markers in combination with multiple cameras to accurately track marker positions [14]. However, this approach has a high cost and requires extensive setup and calibration. Such vision-based methods lack privacy and increase the computational load for processing. Additionally, the patient with PD would require special infrastructure and need to maintain a constant presence within the camera frame, complicating in-home data collection.

A variety of movement measures acquired from various wearable sensors have been utilized to monitor PD movement symptoms. An inertial measurement unit (IMU) is an electronic device that uses a combination of accelerometers, gyroscopes, and magnetometers to measure multi-axis movement acting on it. IMU has been used as a sensing modality to assess movement symptoms in various body sites emphasizing the wrist due to its usage within inexpensive smartwatches and fitness bands [15]. However, this approach has lower precision while measuring fine-grained movement within fingers, unless individual IMU sensors are used for each finger for analysis of specific MDS-UPDRS-III exercises such as finger tapping and hand open-close. Electronic textiles (e-textiles) provide a comfortable and familiar modality to sense and monitor various physiological parameters through the integration of sensors and electronics into clothing. Smart gloves have been proposed by Niazmand et al. [16] for PD movement assessment using a multi-sensor approach. Their sensing glove uses a force sensor placed on the back of the hand, an accelerometer on the middle finger, and conductive fabrics on the thumb and index finger to detect electrical contact associated with finger tapping. The sensing capability of this solution was limited to two types of hand movements (finger tapping and resting hands). Present technology-based movement assessment methods for PD have their own limitations and strengths for remote assessment use cases. Although wearable devices provide a convenient method to carry out such assessments, additional sensors for measuring fine finger movement would be required.

## 3. Materials

This section covers the detailed technical description of our iTex system consisting of sensing gloves, IoT architecture, and the patient-centered user interface.

- Design and Development of iTex Gloves:

Most wrist-worn wearables are typically designed to be worn only in one arm, making them unsuitable to monitor PD symptoms that may appear in both arms. Additional sensors to measure finger flexion are needed to understand anomalies associated with fine-movement tasks. In our study, we propose smart gloves that are worn on both hands while the participant performs certain movement exams.

The wearable computing device on each glove was powered by the ESP32 microcontroller (MCU) with integrated Wi-Fi capabilities. The M5StickC Plus module consisting of the ESP32, rechargeable LiPo battery, 6 DoF IMU was used as a base platform for the glove [17]. The integrated 1.14” color LCD conveys battery percentage, connectivity status, and present movement exam. The core clock speed of the microcontroller was reduced to 160 MHz instead of the default 240 MHz to reduce power consumption. The flexion sensors were interfaced to the analog acquisition board that was connected to the wireless embedded system using the Grove connector. The sensor interface board used a voltage divider circuit comprised of flexion sensors and a 10 kohm (0.5% tolerance) resistor to convert resistance changes associated with finger flexion into voltages for the three analog channels. The pair of gloves aim to provide more information on fine movement movements associated with the finger tapping and closed grip than an inertial sensor mounted at the back of the hand.

**Figure 2:**
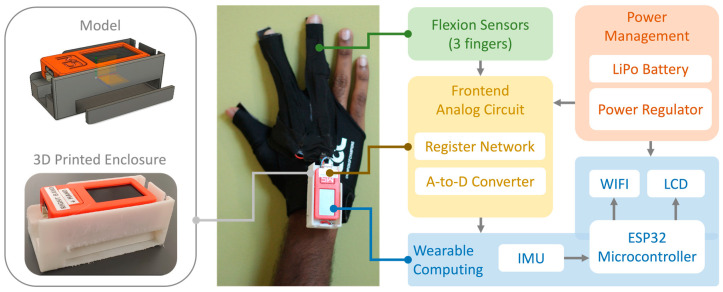
A functional block diagram of the iTex glove.

**Figure 3:**
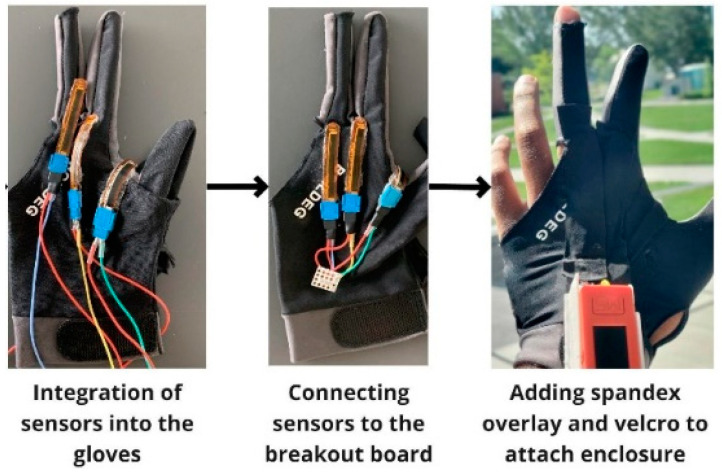
Assembly process for iTex glove.

Specifically, we aimed to measure the movement of the index finger, middle finger, and thumb. To measure finger flexion, the 2″ polyamide-backed resistive flex sensor (Flexpoint) was used [18]. It was found to be appropriate for measuring fine changes in bend angle repeatably due to lower time-varying decay compared to other resistive flexion sensors [19]. Additionally, it provides minimal resistance to flexion and conforms easily to gloves. The position of the bend sensor in the index and middle finger was aligned such that the base of the sensor was over the knuckles as seen in Figure 2. For thumb movement sensing, positioning the sensor directly over the thumb provided minimal variation during finger tapping and closed grip exams. Based on this observation, the sensor was placed between the index finger and thumb to record movement associated with the same movement tasks. The sensors were interfaced directly to a pair of three-finger billiards gloves to improve usability for PwPD who have often had problems coordinating fine movement in their fingers. Figure 3 showcases the assembly process for the iTex glove prototypes. The M5stickc unit and the ADC PCB were housed with a 3D printed enclosure (58.5 × 24 × 28 mm) and weighed 15 g, seen using the FlexFill 98A filament which has characteristics of hard rubber providing impact protection and improves the robustness of the system.

**Figure 4:**
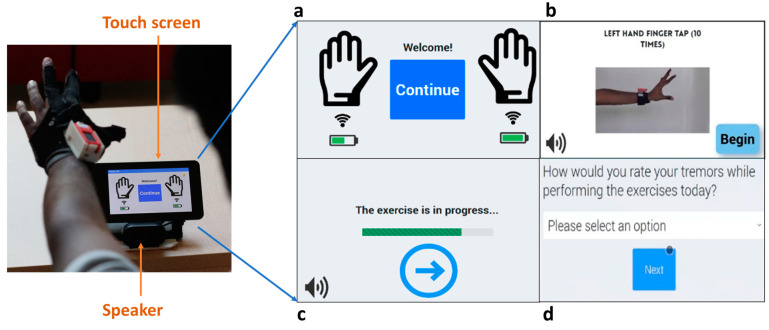
Patient-centered interface: (**a**) Welcome screen with battery status; (**b**) Audiovisual instruction for movement task; (**c**) Movement task timer with the prompt to end task upon early completion; (**d**) Daily symptom questionnaire.

B. IoT Data Collection Architecture:

The gloves were configured to connect to the WiFi access point hosted by the IoT FogNode (Raspberry Pi 4 with touchscreen case) running RaspAP (see Figure 4) [20].

Message Queuing Telemetry Transport (MQTT) over WiFi was used by the gloves to receive movement task activity code start triggers in order to send sensor payloads on demand. The MQTT protocol used a subscribe–publish messaging protocol in any specified topic that was defined as a character array. The devices subscribed to a base activity topic and upon receipt of the activity code payload, a new publish topic was created based on the activity code. Upon receipt of a task, triggers began over MQTT from the client application, and the iTex devices began publishing MQTT payloads containing the sensor values to the FogNode. The sensor payloads were prepared as and when measurements were made by ADC and IMU. The sensor data upon sampling was encoded into a character data array which was then concatenated to the payload substring. When 32 samples were collected for all the IMU and ADC channels, the payload substrings were merged and transmitted over MQTT. This was necessary since the MQTT protocol required payloads to be sent in the Unicode character array format. Sensor payloads were sent using the Async-MQTT client library due to its non-blocking nature associated with its MQTT publish and subscribe methods.

**Figure 5:**
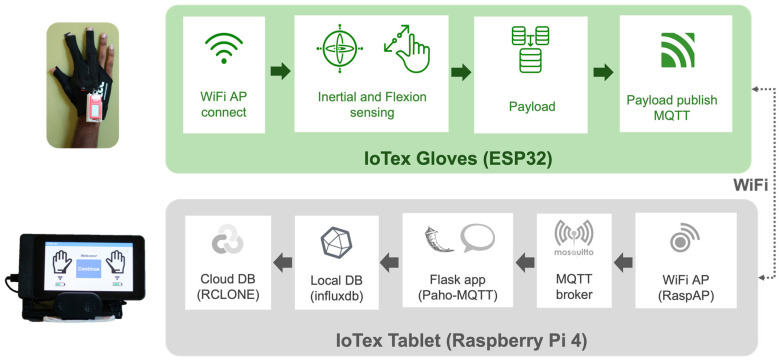
System architecture of iTex glove data collection system.

The topic the gloves published to was determined by glove hand (left/right) and the movement tasks seen in Table 1. Figure 5 shows the sensor acquisition pipeline within the proposed system. The incoming MQTT payloads were handled by the FogNode which runs a Mosquitto MQTT broker service [21] in the background.

C. Design of Patient-Centered User Interface with Accessibility Factors:

The iTex tablet application plays a central role in the system since PwPDs should be able to perform the movement exams on their own independently in unsupervised settings such as homes. Therefore, we designed a patient-centered interface with consideration of accessibility in different ways including bigger icons, color schemes, audio prompts, and simple navigational flow [22]. For example, the interface provides audio-visual prompts to the participant about how a certain movement exam can be performed. It also triggers the MQTT payload transmission for each movement task from the iTex bands and gloves. The touchscreen browser application utilizes the Flask server framework for Python that is hosted locally on the FogNode. Flask was selected for this role due to its seamless integration between the browser and the backend system via Python. The flask application also connects to the local Mosquitto MQTT broker as an MQTT client and subscribes to the topics to which the left and right glove or bands send sensor data payloads.

The Flask application and accompanying web interface were configured to run upon boot in kiosk mode which allowed for it to be in full screen with touch gestures disabled. Application user flow:

1. Welcome screen with device battery status (Figure 4a)
2. Movement task instruction (Figure 4b)
3. Task timer with continue button (Figure 4c)
4. User daily symptom questionnaire (Figure 4d)

The movement task instructions were provided to the participant sequentially for the left and right hand, as listed in Table 1. The “both hands out” and “resting hands” exercises were, however, performed with the left and right hands at the same time for 10 s. After all activities were performed, a daily questionnaire was shown to the participant to obtain self-reported measures of movement symptoms, medication usage time, and sleep status as seen in Figure 4d.

D. Data Logging:

The storage and logging of sensor data from the bands and gloves were handled by InfluxDB within the FogNode client application. InfluxDB is an open-source time-series database system that stores data along with timestamps as key-value pairs that are optimized for usage in high-speed IoT applications [23]. The flask application upon startup begins the InfluxDB service and resets previous exercise tables. The movement task payloads were received as a character array with substrings containing individual sensor data measurements separated by the semicolon character. The individual sensor channel data were then reformatted into a JSON structure and assigned a unique UTC microsecond timestamp and appended to a buffer list for left and right sensor payloads. When the buffer size reached 128, the batched list was written into the InFluxDB database. Within the database, two separate tables were used for left and right band/glove sensor data. Tags corresponding to the present movement task code were also added within the InfluxDB entries to enable easy retrieval of data.

E. Telemetry and Remote Logging:

At the end of all movement tasks, the tables corresponding to the left and right sensor data were exported into a CSV file along with the activity labels and time intervals between the samples. The CSV files exported daily were stored in a folder name corresponding to the present date, and the file names were determined as a combination of hand name and date–time. The daily questionnaire responses were exported into a text file within the same folder. This ensured identifying when the movement tasks were performed and correlating them with the medication usage schedules obtained from the questionnaire. Daily activity folders were synchronized to a cloud storage system using the Rclone utility [24]. Rclone is a command-line program that enables the synchronization, transfer, and mounting of files to cloud storage. An Rclone remote was configured for each participant to a shared cloud folder which was synchronized with the participant data folder.

## 4. Methods

To assess the performance, feasibility, and usability of the iTex system, an in-home study was conducted with four PwPDs. This study was approved by the Institutional Review Board (IRB) at the URI (IRB No: 1715059). The study objective was to evaluate system-level reliability and data quality from the iTex gloves. Additionally, the study was aimed at evaluating the user experience and usability of the proposed system.

- Glove Data Measures

To evaluate the symptoms of tremors, dyskinesias, and bradykinesia in PwPD, fine motor movements are assessed while performing upper-body exercises taken from the third part of the MDS-UPDRS evaluation. In this study, we decided to measure 6 degrees of Freedom in the wrist and three-finger flexion in both arms with our data collection hardware as seen in Table 2. Additionally, we gathered self-reported measures of tremors, dyskinesias, sleep quality, medication intake time, and medication effect from participants daily.

**Figure 6:**
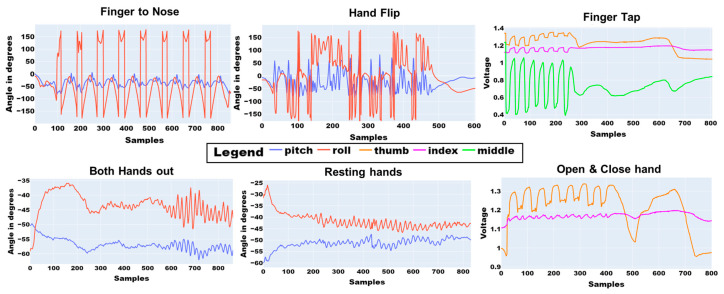
Inertial, flexion signals obtained for the various movement tasks.

The data quality from the iTex gloves (Inertial: pitch, roll and Flexion: index, thumb, middle) as seen in Figure 6, show that the gloves can detect the fine movement tasks associated with the MDS-UPRDS-III. As seen in Figure 6 in the resting hands and holding both hands out sensor data plot, higher frequency components are present within later segments of the data. The plots also indicate that inertial signals (pitch, roll) capture movements associated with Finger to Nose, Hand Flip, Both Hands out, and Resting Hands. Meanwhile, flexion signals (index, thumb, middle) capture movements associated with Finger Tapping and Open and Close hand tasks. Additionally, the data indicate that the kinetic movement task (Finger Tapping) includes resting data since the user might complete the task earlier.

B. Glove Sensor Signal Processing

**Figure 7:**
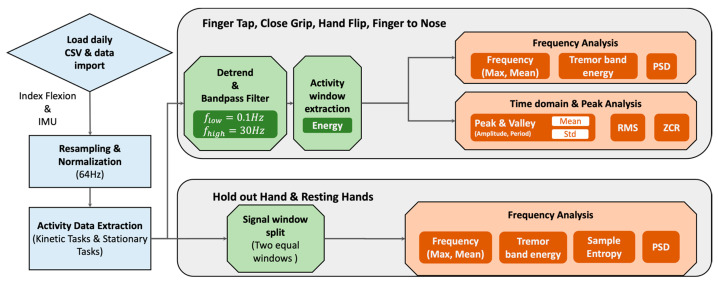
Signal processing pipeline used with iTex gloves.

Signal Processing Pipeline: Figure 7 summarizes the signal processing steps of extracting time and frequency domain features for the different upper body movement tasks. The movement-related flexion and inertial sensing data from sensing gloves were extracted for pre- and post-medication intake for analysis. Essentially, sensor signals from flexion sensors and IMU were resampled at 64 Hz to make the data consistent for advanced processing and analysis. Later, each activity data were extracted from the raw signals.

**Figure 8:**
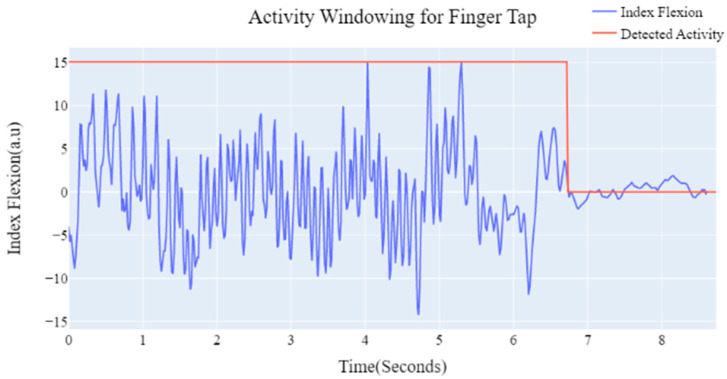
Adaptive activity windowing example for finger tap task.

Adaptive Windowing: For proper analysis, it is important to extract and isolate the activity data before processing. To perform adaptive windowing of the kinetic movement task, we utilized a modified voice activity detector (VAC) concept to detect activity segments [25]. The VAC utilizes sliding window over the sensor data to compute energy within frames and sets adaptive thresholds based on mean energy and minimum energy. These windowed activity sensor data are processed within the signal pipeline based on the type of movement tasks (e.g., index flexion for Finger Tapping; inertial signals for Hand Flip, Finger to Nose, Resting Hands, and Hold Out Hand). Figure 8 shows the example of the activity detector for the finger tapping task; it indicates that the activity detector can segment and isolate sections with motion data.

Feature Set Development: *The* best-performing features reported in the literature include band energy for the tremors (5–10 Hz) and levodopa-induced dyskinesia (2–5 Hz) for resting and kinetic movement tasks [26]. We developed a feature set (detailed in Table A1 (see Appendix A) for the following movement task signals (index flexion, pitch, roll), for kinetic movement tasks (finger tap [ft], hand open-close [oc], finger to nose [fn], hand flipping [hf]), and stationary movement tasks (hold hand out [hh] and resting hands [rh]). For kinetic movement tasks, the task window is split into three equal sections (start, middle, and end) to detect peak and valley amplitude and interval changes associated with PD. The stationary movement task signals were split into two sections when extracting the corresponding features. Features were extracted sequentially for each session across participants. The medication intake labels were grouped into pre-medication (new label: 0, old label: 0–3 h) and post-medication (new label: 1, old label: 4+ h) bins and stored alongside participant, task ID, and features. Later, the extracted features were used in the machine learning classification explained in Section 5.1.

C. Participants and Study Overview:

Four individuals (two male, two female) were recruited to the study with ages ranging from 54 to 86 years. The participants had been diagnosed with PD between 2 and 19 years. The recruited participants had mild–moderate PD symptoms without using a deep brain stimulation implant for this feasibility study. Although this feasibility study does not involve any therapeutic intervention for PwPD, the medication intake time is a key independent variable that affects this study. Participants in the study took their PD medication as per the dosage schedule provided by their physician. They were advised to use the iTex system before and after the medication intake.

As part of the onboarding, participants were requested to take the Montreal Cognitive Assessment (MoCA) test [27]. The test is a brief 30-question test that takes around 10–12 min to complete. The test intends on gauging participants’ cognitive abilities including short-term memory, executive function, language abilities, attention, abstraction, and orientation. The cognitive pre-assessment was necessary to ensure each participant’s ability to provide informed consent for the study. PwPDs in the later stages of the disease might have had mild cognitive impairments that might affect their ability to provide informed consent. If the participant met the MoCA cut-off of 22, they were then provided with the informed consent document and details about the study were provided to the participant. Once the participant provided signed consent, they were asked a few questions about their present Parkinson’s disease treatment plan and medication dosage schedule. This study had no influence on their medication schedule using it as an independent variable. Following initial setup and Wi-Fi connectivity, the first data collection was performed by the participant as a reference video was recorded by the study team to be shared with a trained UPDRS grading expert for assessment of upper body movement tasks. Participants were incentivized by a cash reward and were allowed to withdraw from the study at any time without negative consequences. We conducted an exit interview at the end of the study to understand human factors and constraints around tablet applications. After obtaining consent to record the audio of the interview, it was recorded and deleted after transcription.

## 5. Results and Discussion

**Figure 9:**
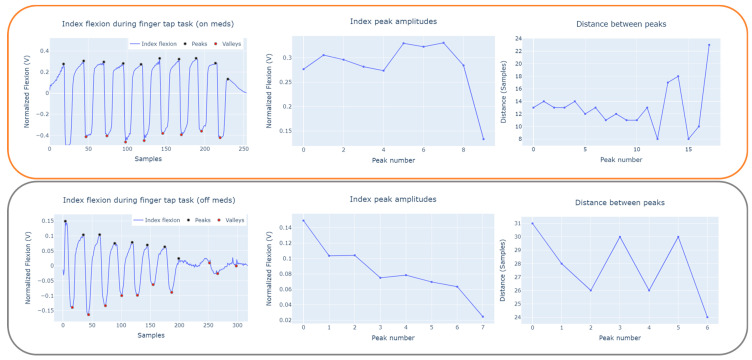
Case plot of right index flexion amplitude and tap interval obtained based on peak-valley analysis for pre- and post-medication states.

In this section, we present and discuss results from the in-home feasibility study measuring the system’s efficacy and usability. We primarily focused on system-level qualitative measurements of performance, usability, and participant adherence. We performed a preliminary investigation on suitable signal features for the movement tasks that correlate with medication intake. We also evaluated the power consumption associated with the MQTT data collection system and the performance of the wireless data acquisition system. We examined the feasibility of using smart gloves capable of performing the remote assessment of fine and gross hand movement tasks within the PwPDs. We found that the features extracted from the kinetic and resting movement signals could reflect a response to Parkinsonian medication. In Figure 9 we show an example of the peak amplitude and intervals based on index finger flexion during the Finger Tap task before and after the intake of medication in Participant 3. We can observe that our iTex gloves can capture the reduction of tapping amplitude and consistency in tapping interval within individuals with PD on and off medications.

### 5.1. System Performance

**Figure 10:**
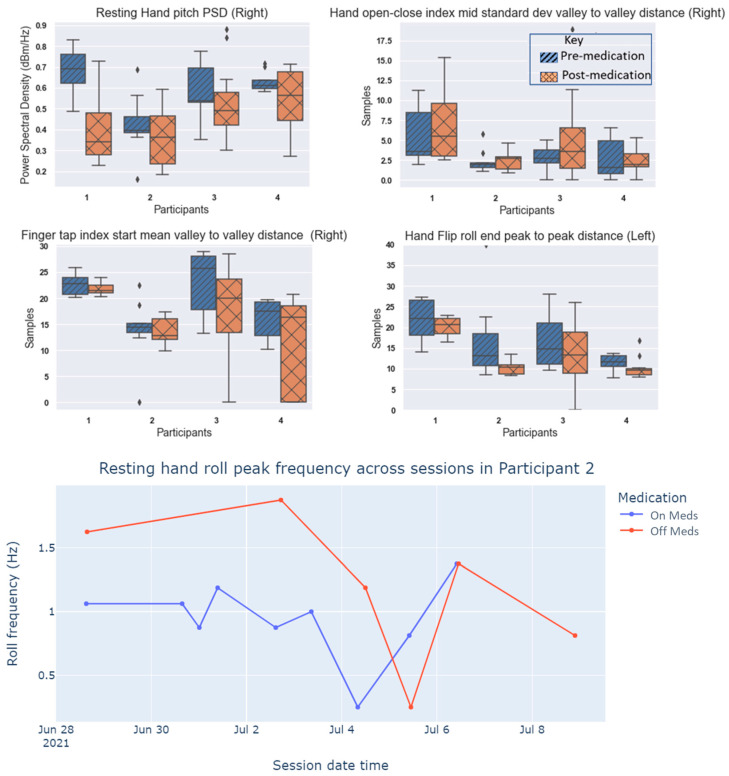
(**Top**) Box plot of features from iTex gloves across participants for pre- and post-medication states. (**Bottom**) Line chart showing resting hand roll peak frequency across sessions for Participant 2.

**Figure A1:**
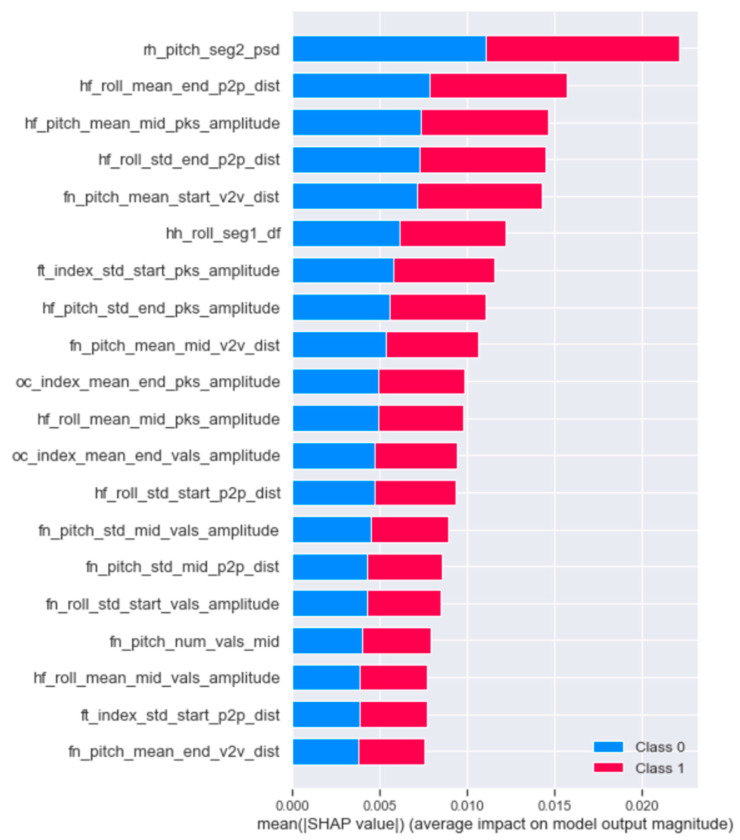
SHAP feature importance for top 20 features from left and right glove data for medication state classification.

Preliminary Machine Learning Classification: To identify the significance of different movement task features, we split the feature dataset without labels into an 80–20 train-test dataset. We then trained a random forest classifier on the train set and provided the tree model to Shapley Additive Explanations (SHAP) to calculate feature importance for the test set (see Figure A1 in Appendix A). Box plots of some of the best-performing features based on medication intake timing for the participants can be seen in Figure 10 aggregated across sessions. The concatenated feature dataset for the left and right gloves were classified using different learning models within sk-learn Python libraries such as K-nearest neighbors, random forest, naïve-Bayes, multilayer perceptron, and support vector machine. The weighted precision, recall, F1-scores, and accuracy associated with each of the models can be seen in Table 3. Stationary task features such as mean gyroscope frequency and pitch dyskinesia band energy show a relationship with medication intake across participants. Meanwhile, kinetic movement task features such as mean frequency and peak-to-peak distance generally performed better on the non-dominant hand (left) for participants, with considerable variability across participants in relation to medication intake. This highlights the importance of conducting a holistic analysis of features through different assessment tasks to account for the variability in symptoms among the participants. The choice of learning model seems to affect the classifier performance with random forest performing best followed by multilayer perceptron and support vector machine for the merged feature dataset.

MQTT-based wearable sensor data collection performance: To evaluate the performance of the wireless data acquisition system, we evaluated the effective sampling rate from the FogNode logging system for the fixed-time movement tasks (Both Hands Out, Resting Hands). The average effective sampling rate of the proposed system is reported in Table 4 for different participants. Investigation of the sampling rate revealed a bottleneck within the acquisition pipeline, considering the glove data is sampled at 128 Hz. During the data rate assessment, we identified the effective data rate was lower than the target 128 Hz, specifically when both gloves were streaming. To identify the source of this bottleneck within the pipeline, a timing test was conducted to evaluate the time taken for different operations for an entire session and averaged, as seen in Table 5.

The findings from the timing tests indicate that the lowering of the effective sampling rate was caused by the high inter-payload interval within the MQTT protocol, rather than the payload parsing or database operations. The feasibility study leveraged the session-based glove data export to the cloud for conducting data quality assessments remotely. COVID-19 imposed logistical challenges that prevented hardware maintenance for gloves. The participants were, however, provided support for software issues with the tablet through remote shell-based debugging on demand. The cloud data export was reliable and worked as expected with all participant sessions. Even in cases where there was no internet during a session, the missing data was uploaded alongside new data. Upon investigation, we identified that the MQTT client was overwhelmed by the data rate within the same script. This, however, can be easily resolved by converting the application architecture into multiple asynchronous sensor data handler scripts and by reducing the data rate.

**Figure 11:**
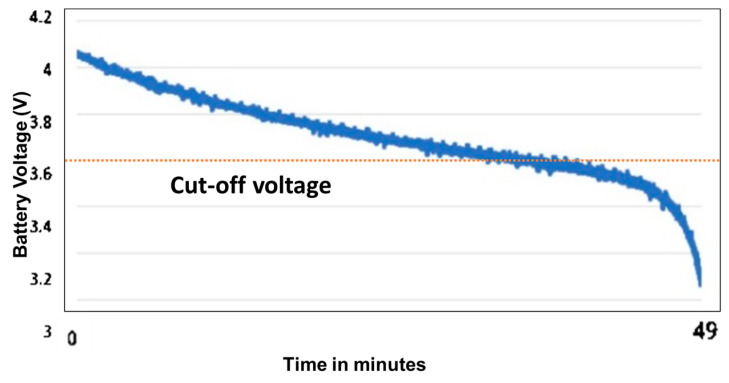
Battery discharge curve of iTex glove during continuous wireless transmission (Battery cut-off voltage set to 3.7 V in AXP192).

Power Consumption Test: The power consumption of the proposed iTex glove is analyzed by recording the battery voltage. The battery voltage is measured by the onboard power management integrated chip (AXP192). For this battery discharge test, the glove was set to publish the sensor payload continuously over WiFi using MQTT to iTex tablet at a range of 1 m. The battery discharge curve obtained from the test is shown in Figure 11. Using the last published MQTT message prior to the glove turning off, the battery life for continuous payload transmission was found to be 35 min in a single run which is acceptable since our exam typically takes around 5–10 min for the user to complete.

### 5.2. Usability Results

**Figure 12:**
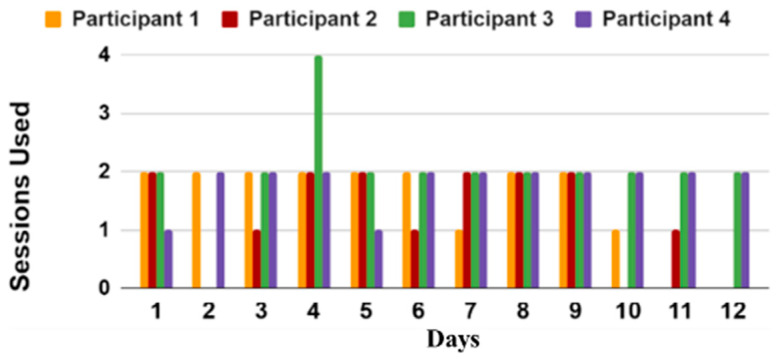
Bar graph representing participant adherence in using iTex system.

Participant Adherence: We investigated the participants’ adherence to using the iTex system as an indirect measure to understand the feasibility of this system. The adherence of participants using the iTex system was evaluated by analyzing the data logs and extracting the time and date for each session. The resultant sessions were grouped by day and visualized using a bar graph as seen in Figure 12. It was found that participants used the iTex system typically two times a day as instructed. There were certain days when participants did not use the system at all and days when they used the system only once. Participants 3 and 4 used the system most consistently, with Participant 3 using the system four times in one day. Participants 1 and 2 did not perform the test consistently in the last few days of the period.

Table 6 shows the usability measures for different aspects of the iTex software application. Although all participants found the application to be straightforward to use, some participants felt the questionnaire page was moderately difficult to use with their tremors. When the participants were asked if their experience using the application was affected by their medication status, most participants reported they had not noticed any difference.

**Figure 13:**
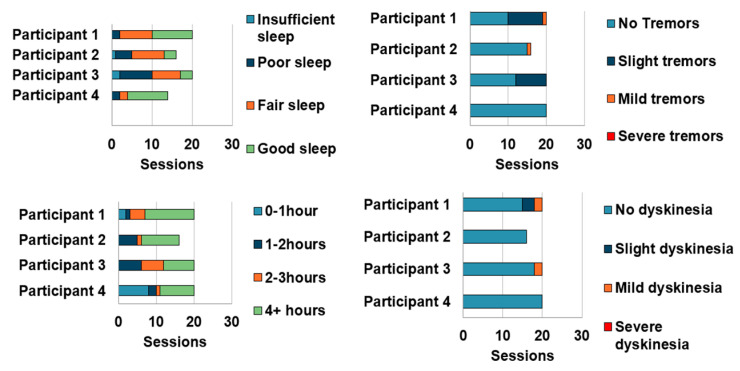
Bar graph representing self-reported measures obtained through daily questionnaire (top-left) sleep quality, (top-right) tremor symptom, (bottom-left) medication intake time, and (bottom-right) dyskinesia symptom.

As seen in Table 7, Participants 3 and 4 found that their gloves did not properly fit them and found the gloves difficult to put on. All participants found the magnetic charging cable to be helpful to charge the gloves system. Participant 3 reported that he found the number of cables for powering tablets and the gloves to induce more stress and wondered if better cable management of wireless power could be used in the future. From the self-reported measures in Figure 13, we can see that participants primarily reported slight and mild tremor and dyskinesia symptoms. The participants also utilized the system under different medication intake periods. We were able to identify certain usability issues participants faced with the tablet application and the gloves. Multiple participants had reported that they found the questionnaire response selection drop-down to be difficult to use particularly while off medication. The glove-wearing and removal process was reported to be tedious by certain participants particularly when they were off-medications. Fit issues were also present with participants’ larger hand sizes. These highlight the need for personalized gloves and incorporating usability-enhancing design mechanisms.

Although Participant 1 felt the gloves were difficult to wear after intake of medication, Participants 2 and 4 found no difference.

## 6. Conclusions

In this work, we presented the iTex system, which is composed of sensor-equipped gloves and a tablet application that provides movement task prompts based on upper body UPDRS Part III exams to facilitate the in-home assessment of PD and remote logging. Our design enables the sensing of inertial motion signals and finger flexion (middle, index, and ring) along with daily questionnaires that were aimed to measure self-reported PD symptoms. We have also shown initial results that indicate variations induced by PD medication usage. Future hardware revision of the embedded system can see the integration of the microcontroller, wireless unit, and ADC board into a single PCB. Additionally, a 9-DOF IMU can be used instead of a 6-DOF IMU to obtain accurate yaw data from the hardware. Through evaluation of the wireless data acquisition system, we identified a bottleneck associated with the MQTT transmission, resulting in the reduction of the effective sampling rate. This problem can be addressed in a multitude of ways from reducing sampling rate (studies have reported 50 Hz is sufficient for PD assessment [28]) to optimizing payload size and through the use of lightweight MQTT-SN protocol (Utilizes UDP instead of TCP/IP). Future data collection efforts can include age-matched controls and add PwPD with mild–severe symptoms to obtain a comprehensive understanding of PD symptom metrics.

Additionally, in this work, we relied on self-reported measures of medication intake. However, this is imprecise and can introduce variability during analysis. Accurate and objective logging of medication intake time and physical exercise is required to obtain meaningful insights from the features extracted from the iTex sensor data. In the future, we plan to extend the study with more PwPD with an improved protocol to increase the reliability of the medication intake data. Additionally, evaluation of the test–retest reliability of the iTex system can be conducted to study the influence of other contextual variables (environmental, physical activity, and cognitive stressors).

## Figures and Tables

**Table 1:** Movement task instruction details.

Movement Task	Task Instruction
Finger Tapping (FT)	Tap the index finger on the thumb 10 times as quickly AND as big as possible.
Open and Close Hand (OC)	Open the hand 10 times as fully AND as quickly as possible.
Hand Flip (HF)	Extend the arm out in front of the body with the palms down, and then turn the palm up and down alternately 10 times as fast and as fully as possible.
Both Hands Out (HH)	Stretch the arms out in front of the body with palms down for 10 s.
Finger to Nose (FN)	Point your left index finger towards the screen, then bring your index finger to your nose and point it out again 10 times as quickly and steadily as possible.
Resting Hands (RH)	Sit in a chair and rest your arms on your armrests or on your thighs for 10 s.

**Table 2:** Data measures associated with iTex gloves.

Data Measures	Channel	Sensor	Sampling Rate
Flexion sensing	Index, thumb, ring	Flexpoint (resistive)	128 Hz
Inertial sensing	Wrist acceleration, gyroscope [AHRS computed]	MPU6886 (MEMS IMU)	128 Hz
Power usage	Battery discharge voltage, current	AXP192	16 Hz

**Table 3:** Classification of different learning models for feature datasets.

Model	Precision	Recall	F1-Score	Accuracy
K-nearest neighbors	0.69	0.65	0.63	0.65
Random forest	0.72	0.71	0.71	0.71
Naïve-Bayes	0.67	0.59	0.53	0.58
Multilayer perceptron	0.71	0.68	0.67	0.68
Support vector machine	0.69	0.65	0.63	0.66

**Table 4:** Average effective sampling rate of the system.

Participant	Left Glove (Hz)	Right Glove (Hz)
1	82	84
2	84	84
3	82	83
4	87	86

**Table 5:** Timing involved with different operations within the FogNode client application.

Operation	Payload Parsing	JSON Formatting	InfluxDB Write	Inter Payload Interval
Time taken (ms)	0.296 ± 0.03	1.71 ± 0.4	13 ± 15	364 ± 23

**Table 6:** Application usability responses.

Question	Participant 1	Participant 2	Participant 3	Participant 4
Ease of use	Very Easy	Easy	Very Easy	Very Easy
Clarity of instruction	Easy	Very Easy	Very Easy	Very Easy
Preferred time to use application	Morning and afternoon	Morning	Morning	Morning
Application usability and medication use	Did not affect	Did not affect	Hard to select questionnaire responses when medicated	Did not affect
Time spent per session	7 min	5 min	8–15 min	10–15 min

**Table 7:** Glove usability responses.

Question	Participant 1	Participant 2	Participant 3	Participant 4
Ease of wearing [1 = Difficult, 10 = Effortless]	8	9	4	7
Fit of gloves [1 = Improper fit, 10 = Perfect fit]	10	8	4	7
Ease of charging [1 = Difficult, 10 = Effortless]	10	10	10	9

## Data Availability

Data will be made available on request in 2024.

## Abbreviations

PD	Parkinson’s disease
PwPD	People with Parkinson’s disease
IMU	Inertial measurement unit
DOF	Degrees of freedom
MDS-UPDRS	Movement Disorder Society Unified Parkinson’s Disease Rating Scale
IoT	Internet of Things
PT	Physical therapy
ADC	Analog to digital converter
MQTT	Message Queuing Telemetry Transport
UTC	Universal Time Coordinated
PSD	Power spectral density
RMS	Root mean square
ZCR	Zero crossing rate
AU	Arbitrary units

## Appendix A

**Table A1:** Description of features extracted from iTex glove data.

Type of Task	Movement Task	Signal Type	Features Extracted
Kinetic Task (start, mid, end)	FT	Flexion	Mean frequency (mf) Max frequency (df) Band energy ○ Tremor (pdenergy) ○ Dyskinesia (dysenergy) Power spectral density (psd) Mean peak–peak interval (p2p_dist) Std peak–peak interval (p2p_std) Mean valley–valley interval (v2v_dist) Std valley–valley interval (v2v_std) Mean peak amplitude (mean_pks_amplitude) Std peak amplitude (std_pks_amplitude) Mean valley amplitude (mean_vals_amplitude) Std valley amplitude (std_vals_amplitude) Root mean square (rms) Zero crossing (zcr)
	OC	Flexion	
	HF	Inertial	
	FN	Inertial	
Stationary Task (start, end)	HH	Inertial	Band energy ○ Tremor (pdenergy) ○ Dyskinesia (dysenergy) Sample Entropy (sent)
	RH	Inertial	
